# A Rare and Urgent Consequence After a Snake Bite

**DOI:** 10.7759/cureus.21910

**Published:** 2022-02-04

**Authors:** Carolina Lizarzaburu-Ortiz, Gabriela Yumi, Andrea Carvajal, Ana B Pachacama, Alexandra Berrazueta, Eduardo Rojas

**Affiliations:** 1 Plastic and Reconstructive Surgery, Military Hospital, Quito, ECU; 2 Infectious Diseases, Military Hospital, Quito, ECU; 3 Medical School, Universidad Internacional del Ecuador, Quito, ECU

**Keywords:** serum sickness, fasciotomy, compartment syndrome, venom, snake bite

## Abstract

Snake bites (ophidism) constitute a public health problem in tropical and subtropical countries, due to their lethality, and the lack of access to prompt treatment in these regions. Catastrophic consequences follow the injection of the venom due to its proteolytic, procoagulant, hemorrhagic, nephrotoxic, vasculotoxic, and myonecrotizing properties, which can lead to severe complications if not managed promptly and adequately. In this report, we present the case of a 26-year-old male patient who suffered a snake bite (Bothrops asper) on the back of his right foot. Initially, he received eight vials of antivenom and was transferred to a specialized center. However, a few hours after his arrival, a necrotic wound developed on the back of the foot along with compartment syndrome, and hence emergency fasciotomies had to be performed and the patient was admitted to the ICU for multidisciplinary management and continuous monitoring. After 14 days, fasciotomy closure and eschar incision on the dorsum of the foot were performed along with an aesthetic reconstruction with an advancement flap and full-thickness graft. The patient had a satisfactory outcome thanks to the prompt evaluation and access to a unit with experience in managing highly complex cases. Our case highlights the importance of prompt and meticulous assessment after a snake bite due to its potential for causing highly complex clinical scenarios, and early diagnosis, proper management, and continuous multidisciplinary monitoring are key for a favorable prognosis. In many cases, these bites can lead to significant tissue loss and may necessitate the amputation of the affected limb. In severe cases, compartment syndrome frequently ensues and further complicates the management and increases morbidity if not recognized and managed promptly.

## Introduction

**Figure 1:**
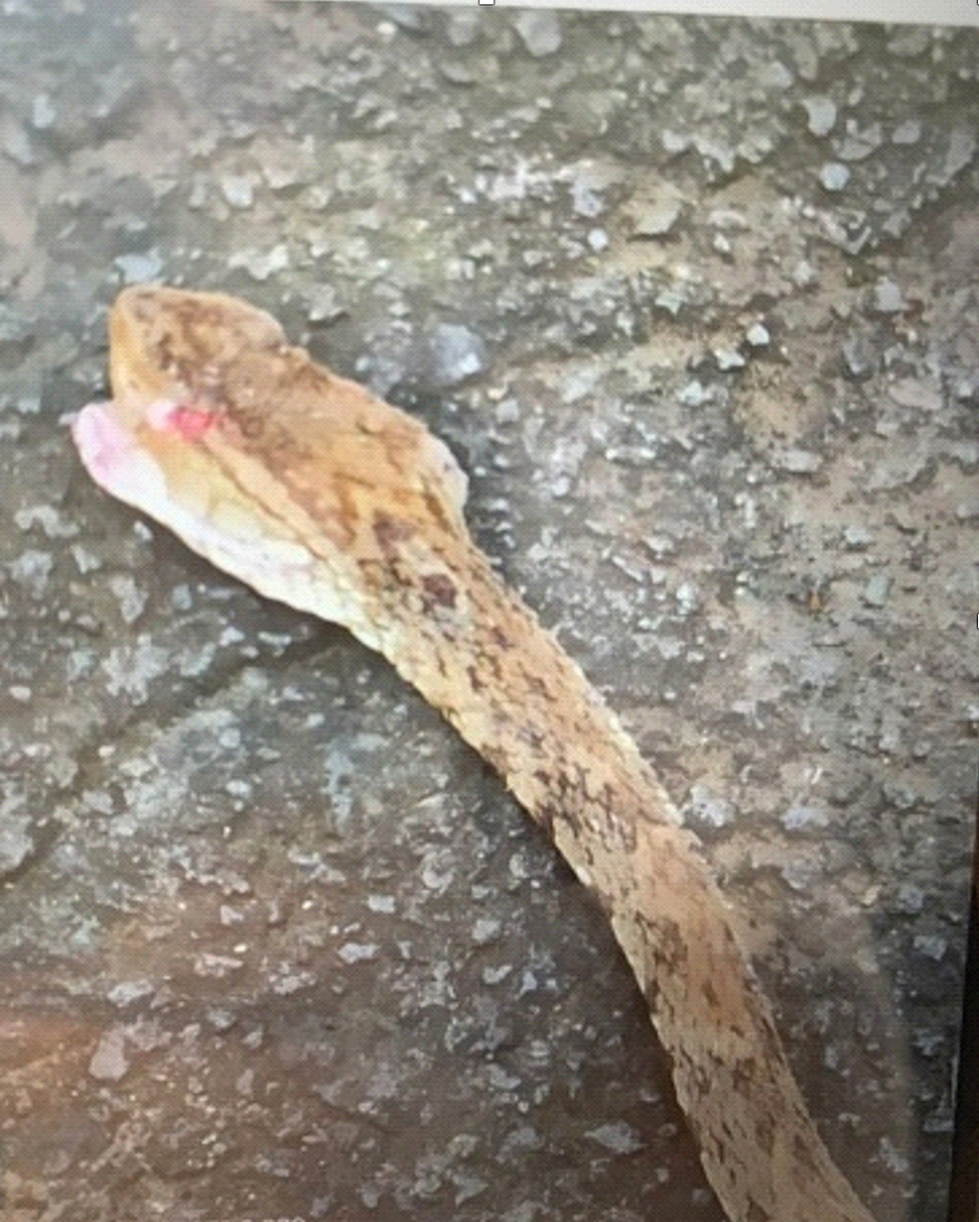
Snake that bit our patient (genus Bothrops of the family Viperidae)

Ophidism is the syndrome caused by the inoculation of poisonous substances through the bite of a venomous snake [1]. It represents a public health problem in tropical and subtropical countries, since the lack of knowledge regarding its etiology and treatment, as well as the application of inadequate curative measures, leads to delays in prompt management [2]. In Ecuador, snakes of the genus Bothrops (Figure 1) of the family Viperidae (Bothrops asper in the western region and Bothrops atrox in the eastern region) are responsible for the highest number of snake-bite accidents (70-80%) [2]. The amount of poison inoculated by a Bothrops asper (velvet or yellow beard) is larger than that by other species, thereby often causing fatal consequences [3]. The complexity of the bite depends on many factors, including the amount of poison inoculated, the affected anatomical site, wound size, size of the snake, and the age of the patients and their general physiological state [3-4].

The poison consists of several toxic peptides and proteins, such as myotoxins, anticoagulant toxins, neurotoxins, and coagulant toxins [4]. In the genus Bothrops, the venom has proteolytic, coagulant, hemorrhagic, nephrotoxic, vasculotoxic, and myonecrotizing properties [5-6], which induce local and systemic manifestations such as severe pain, heat, flushing, hypotension, edema, fever, systemic bleeding, and necrosis [2-3,7]. In bothropic poisoning, manifestations at the local level are associated with the presence of phospholipases A2, metalloproteinases, vasoactive peptides, and serine proteinases that initially induce histamine release and increase capillary permeability [2]. The alterations in the skin are mainly due to the action of metalloproteinases that affect the dermis-epidermis interface, causing the separation of the epidermis and the formation of phlyctena and bullae or blisters, which can lead to ulceration, dermonecrosis, and myonecrosis [2-3]. Systemic manifestations are not frequent but can be severe; bleeding is one of the most common manifestations, leading to hypovolemia, hypotension, shock, disseminated intravascular coagulation, rhabdomyolysis, and renal failure [8].

In addition, complications such as compartment syndrome, although very rare, may occur. Snake venom causes muscle necrosis by itself and produces the extravasation of interstitial fluid or bleeding within the fascial space generating the increase of pressure; however, this manifestation is exceedingly rare and such syndrome has only been reported in 7% of patients and in bites of larger snakes [1]. Furthermore, poisons are highly contaminated with a large number of bacteria [3] and various germs have been found in infected wounds, such as Gram-negative aerobic bacilli (Morganella morganii, Escherichia coli, Proteus rettgeri, Klebsiella spp., Enterobacter spp., Enterococcus faecalis, Aeromonas hydrophila, Pseudomonas aeruginosa, Acinetobacter spp.), strict anaerobes such as Clostridium spp., and a lower proportion of Gram-positive cocci: Staphylococcus epidermidis, Staphylococcus aureus, and Streptococcus spp. [4].

## Case presentation

**Figure 2:**
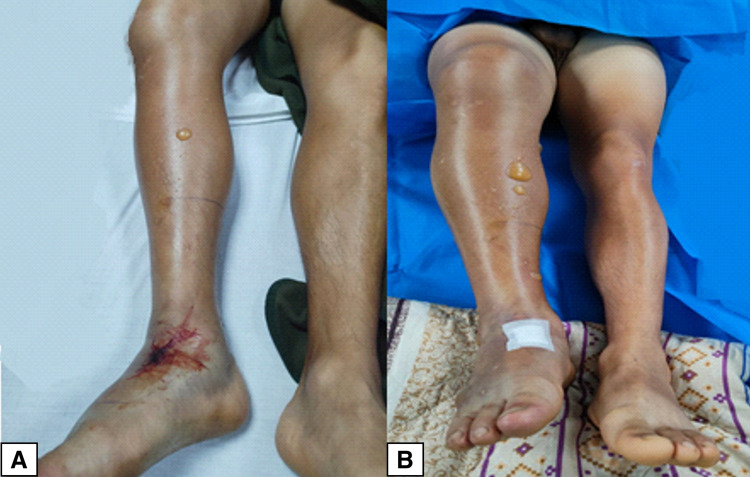
Physical examination after the incident (A) Condition of the leg right after the snake bite located on the dorsum of the right foot. (B) Appearance two hours after the patient's arrival at our center. Note the significant edema and increased number of bullae

**Figure 3:**
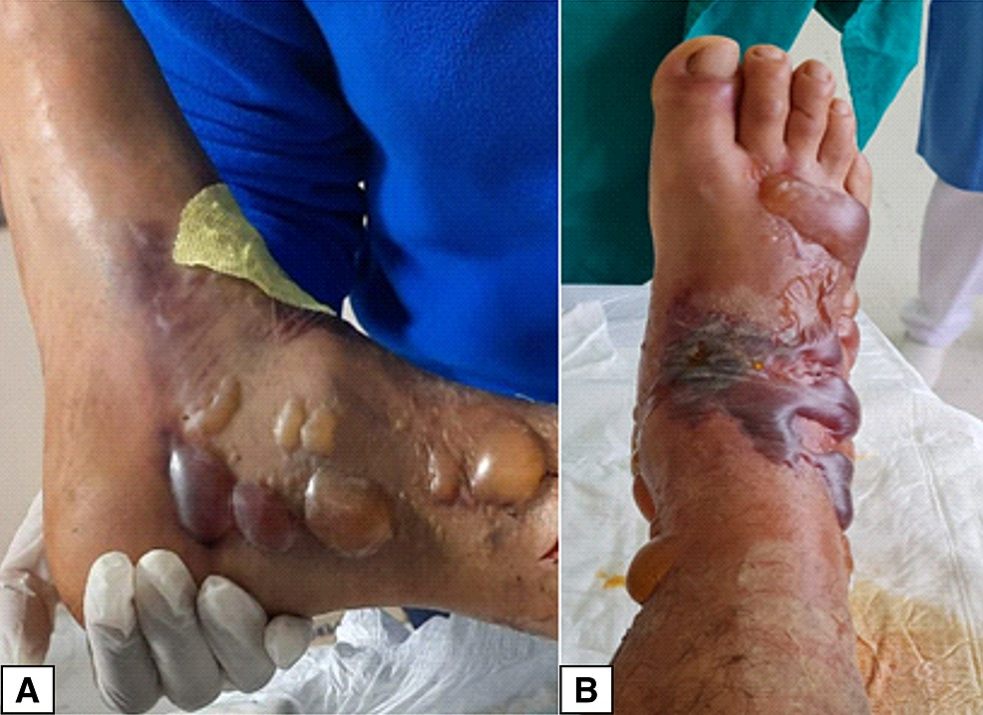
Fast evolution of skin lesions and edema (A) Lateral view and (B) superior view showing multiple bullae and blisters with serous and hemorrhagic content as well as significant soft-tissue edema

We present the case of a 26-year-old male patient who suffered a bite from a Bothrops asper snake (Figure 1) on the back of his right foot in the coastal region of Ecuador (Mataje-Esmeraldas). He received first aid at a local hospital where eight units of antiophidic serotherapy were administered and then he was transferred to our unit (The Armed Forces Hospital No.1) in Quito for specialized and multidisciplinary management. On arrival, he was in severe pain, and there was significant edema in the right lower limb. Laboratory exams showed prolonged coagulation times, PT of 17.7 seconds (normal range: 10.9-14.2 seconds), elevated fibrinogen of 424 mg/dL (normal range: 150.0-400.0 mg/dL), creatine phosphokinase (CPK) of 1,922 U/L (normal range: 0-170 U/L), creatine kinase MB (CK-MB) of 54.3 U/L (normal range: 0.0-25.0 U/L), plus evidence of clinical signs of compartment syndrome in the right leg (Figure 2): absence of distal pulses, paresthesia, and foot cyanosis. Skin lesions evolved quickly and resulted in the appearance of multiple blisters and bullae with a significant increase in the size of the limb compared to the initial assessment (Figure 3).

**Figure 4:**
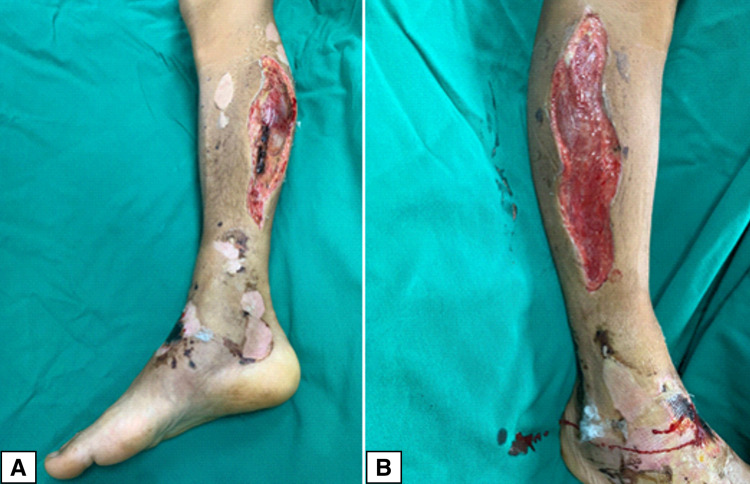
Fasciotomies for compartment syndrome pressure relief (A) Medial fasciotomy with visible necrotic tissue. (B) Lateral fasciotomy

The diagnosis of compartment syndrome necessitated an emergency surgical procedure consisting of both medial and lateral fasciotomies in order to relieve the pressure; during the surgery, necrotic tissue was observed and removed (Figure 4).

**Figure 5:**
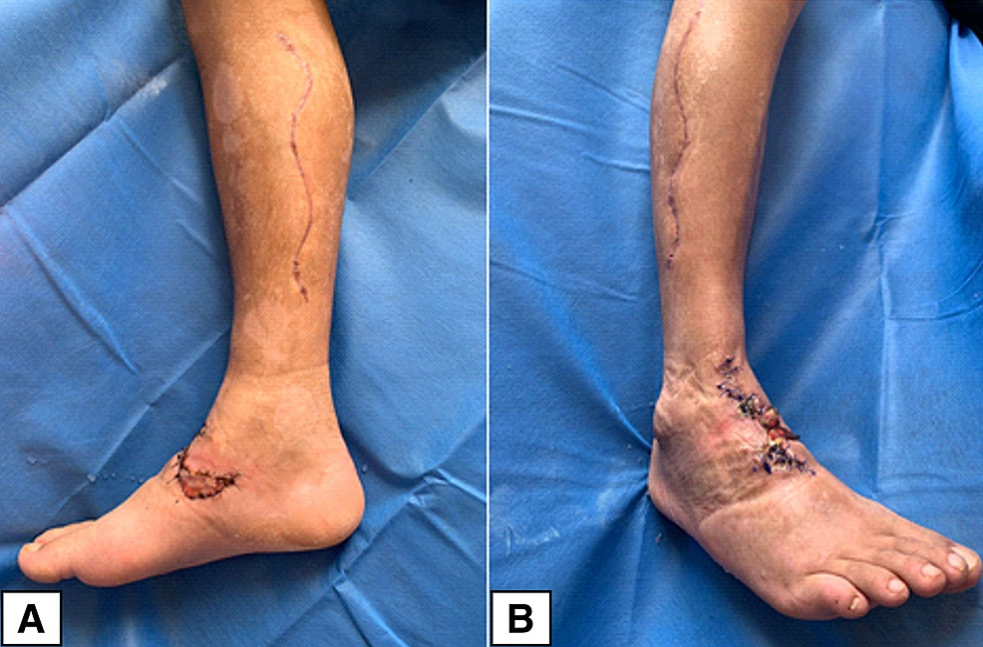
Esthetic results after the closure of fasciotomies and wound reconstruction (A) Closure of medial fasciotomy. (B) Closure of lateral fasciotomy. Note also the reconstruction on the bite site

Afterward, the patient was managed in the ICU, receiving antiophidic serum and antibiotic therapy with clindamycin and cefepime for six days. The patient's vital signs had remained stable since the beginning of his treatment at our hospital and remained so during his stay in the ICU. On day seven, he developed a fever, rash, urticaria, and arthralgia related to antibiotic administration. Therefore, serum sickness secondary to the use of antiophidic serum and triggered by antibiotics was suspected and confirmed [9-10]. The patient continued to receive advanced wound management for his fasciotomies until the 13th postoperative day when closure and repair were performed. Additionally, an escharotomy was performed on the dorsum of the right foot (bite site), which led to the exposure of the extensor tendons and the need for wound reconstruction with a Limberg and forward flaps (Figure 5). A culture of the wounds yielded no growth and the patient resumed his antibiotic therapy with piperacillin + tazobactam after proper desensitization for five days. Subsequently, he developed necrosis in the bite area, and hence a flap and a full-thickness graft were used for a second reconstruction.

At the time of discharge, the patient had no neurological, functional, or systemic sequelae. However, three months later, the patient complained of joint pain and had positive markers for rheumatoid arthritis. This prompted management with a course of corticosteroids, which led to favorable results.

## Discussion

Snake bites are a medical emergency with varying degrees of severity that are influenced by multiple factors such as the affected individual's physiology and comorbidities as well as the biology of the attacking snake. Our case was classified as severe snake poisoning, characterized by the following features: incoagulable blood sample, prolonged prothrombin time, tachycardia, tachypnea, diaphoresis, evidence of compartment syndrome in the right leg corroborated by intense pain, distal cyanosis, multiple blisters and bullae, lower limb edema, and pulselessness. Emergency lateral and medial fasciotomies were performed to decompress the three compartments in the extremity. A patient with severe poisoning should ideally receive 12 vials of antiophidic serum dissolved in 250 ml of saline [2-6]. However, our patient received only eight vials at the first point of care, which presumably failed to sufficiently neutralize the venom, leading to further tissue destruction and the development of compartment syndrome. Therefore, the degree of poisoning severity must be properly determined for adequate management with the antiophidic serum. A proper assessment of clinical signs and symptoms is useful in determining the degree of severity, as described in Table 1.

**Table 1:** Ophidic accident: degrees of severity Classification of ophidic accidents caused by snakes of the family Viperidae based on degrees of severity [2-3]

Parameters	No poisoning	Mild poisoning	Moderate poisoning	Severe poisoning
Injury	No local signs and symptoms	Local edema of 1 or 2 segments. Diameter of the affected area <4 cm with or without ecchymosis. Little or no bleeding	Edema from 2 to 3 segments. Diameter of affected area >4 cm. Ecchymosis with local bleeding. Rarely causes blisters	Head or neck bites. Edema involves more than 3 segments. Compartment syndrome is present. Areas of local necrosis and blisters
Pain	Mild	Mild	Moderate	Severe
Clot test	Coagulates	Coagulates	Does not coagulate	Does not coagulate
Systemic manifestations	No systemic alterations	No systemic alterations	Systemic alterations such as mild hypotension and gingivorrhagia	Severe hypotension with bleeding in several body regions. Can present with disseminated intravascular coagulation (DIC). Can have acute renal or multiorgan failure

Treatment based on degrees of severity

In Ecuador, the most commonly available quantity of antiophidic serum is 10 ml, which is capable of neutralizing between 25-30 mg of Bothrops asper venom [2]. Treatment is determined according to the severity of the condition, which is classified as no-poisoning, mild, moderate, and severe poisoning as described below [2-6].

No Poisoning

Observation is recommended for six hours with a repeated clot test. If the sample coagulates and the edema does not progress, the patient is discharged after counseling about potential warning signs (active bleeding, increased edema, blisters, and ecchymosis) [2-6].

Mild Poisoning

In this scenario, the objective is to neutralize 100 mg of inoculated poison, and at least four vials of antiophidic serum dissolved in 250 ml of 0.9% saline are administered in continuous infusion for 30 minutes [2-6].

Moderate Poisoning

The aim in such cases is to neutralize 200 mg of poison inoculated with the administration of eight vials of antiophidic serum dissolved in 250 ml of 0.9% saline in continuous infusion for 30 minutes [2-6].

Severe Poisoning

The goal here is to neutralize at least 300 mg of inoculated poison with the administration of 12 vials of antiophidic serum dissolved in 250 ml of 0.9% saline in continuous infusion for 30 minutes. The patient should be transferred to a center that has the means to manage highly complex cases and should remain under continuous monitoring [2-6]. Twelve hours after serum administration, a correction of coagulation tests, cessation of local or systemic bleeding, and improvement of the general condition should be evidenced. If the parameters and clinical manifestations do not improve, an additional dose of five or 10 vials of antiophidic serum should be administered [3].

It should be noted that in our case, the lack of proper means to manage the patient at the first center prompted the need to transfer him to our hospital within 10 hours of the incident. This fast referral probably decreased the risk of amputation and guaranteed continuous monitoring in the ICU, thereby also preventing multiorgan compromise. Multidisciplinary management, particularly of such complex cases, is necessary in order to ensure an adequate recovery.

As mentioned in the introduction, snake bites can lead to multiple complications both locally and systemically. However, their treatment is not devoid of adverse events, and a high incidence of serum sickness has been reported, which appears between 7-14 days after the administration of the antiophidic serum [8-10]. Serum sickness consists of a type III hypersensitivity reaction, produced by the deposit of circulating immune complexes (antigen-antibody) in the blood vessels, which activate the complement system, resulting in the release of mediators from mast cells and neutrophils chemotaxis. This, in turn, activates enzymes and destructive free radicals that damage the vessels [9]. Of note, 95% of patients present with fever, maculopapular rash, measles-like urticaria, often pruritic and symmetrical [9]. Initially, it appears on the hands, feet, and thorax, and can spread throughout the body [9]. The first manifestations after the administration of the antiophidic serum may occur at the injection site, such as inflammation of small cutaneous vessels, changes similar to an erythema multiforme (mucous membranes are not involved), palmar erythema, and atypical maculopapular changes on the lateral surfaces of the fingers and toes or alongside the outer edge of the soles of the feet [8-10]. If not treated properly, serum sickness can trigger an anaphylactic shock and even death. In our case, it was necessary to use corticosteroids and antihistamines (methylprednisolone and loratadine) as well as suspend all types of antibiotic therapy, and then desensitization was carried out by the allergology service. In addition, given the high risk of infections, despite having a negative culture, piperacillin/tazobactam administration was completed.

Another important complication is the development of compartment syndrome, which is a rare but not unexpected occurrence. Therefore, knowledge of its signs and symptoms is of paramount importance in order to prevent severe sequelae that occur if the condition is left untreated. Increased intracompartmental pressure reduces capillary perfusion and leads to tissue ischemia [11], which in turn results in irreversible muscular and nervous damage [12]. Despite advances in laboratory and imaging tests, the diagnosis of compartment syndrome is still based on two fundamental pillars: clinical scenario and pressure measurement [12]. The initial suspicion is based entirely on the clinical characteristics: global pain in a segment of the limb disproportionate to the injury, swelling, feeling of resistance to palpation, paresthesias, cold extremity, and absence of pulses [12]. Surgical treatment requires fasciotomies on the affected compartments; in the case of the leg, three anatomical compartments exist, the lateral, deep posterior, and superficial posterior compartments [12-13]. From the surgical point of view, the appearance of an acute compartment syndrome forces the surgeon to make decisions quickly to reduce intracompartmental pressure and thus avoid the dreaded muscular and nervous ischemia [1]. In order to release all compartments, both a medial and lateral fasciotomy should be performed. The medial fasciotomy is used to release the posterior compartments and is performed by cutting from the posteromedial subcutaneous edge of the tibia and extending it from the tibial tuberosity to the musculotendinous junction of the Achilles tendon [12-13]. Finally, the lateral fasciotomy decompresses the anterior and lateral compartments and extends along the length of the leg, 4 cm below the head of the fibula to the myotendinous junction of the fibula; utmost care should be exercised in order to not damage the superficial peroneal nerve [12-13]. In our case, the rapid evolution of soft-tissue edema led to the need for bilateral fasciotomies only two hours after the patient's arrival at our hospital (Figure 4).

Finally, resolution of the fasciotomies and associated injuries is as vital as acute management. The psychological toll of the attack can be further complicated by the constant reminder of the event due to a highly visible and deforming scar, which is why multidisciplinary management with plastic surgery is necessary in order to ensure the best esthetic results. Our patient presented local necrosis in the area of the bite thanks to the aforementioned proteolytic and myonecrotizing action of the venom. This necessitated an escharotomy and closure by means of a rotation flap and full-thickness graft placement with adequate esthetic results (Figure 5).

## Conclusions

A snake bite should be treated as a medical emergency as it requires antidote administration (antiophidic serum) within the first 24 hours in order to prevent worsening complications. To achieve this, antiophidic serum should be administered based on the degree of severity (more severity, more doses), resulting in a reduced rate of necrosis and amelioration of the risk of death. In compartment syndrome, fasciotomies are a mandatory part of the treatment and must be performed without delay to prevent necrosis of the limb. However, it must only be performed once the diagnosis is suspected and not as a prophylactic procedure to prevent the development of compartment syndrome in patients suffering from an ophidic accident. Finally, our case highlights the importance of multidisciplinary management in high-complexity scenarios, including the benefits of bringing in multiple specialists such as plastic surgeons. The prognosis of such patients is influenced by multiple factors, but delay in the administration of specific serotherapy and management in non-specialized units can lead to catastrophic consequences.
